# Non‐genomic rewiring of vitamin D receptor to p53 as a key to Alzheimer's disease

**DOI:** 10.1111/acel.13509

**Published:** 2021-11-02

**Authors:** Rai‐Hua Lai, Yueh‐Ying Hsu, Feng‐Shiun Shie, Che‐Ching Huang, Mei‐Hsin Chen, Jyh‐Lyh Juang

**Affiliations:** ^1^ Institute of Molecular and Genomic Medicine National Health Research Institutes Zhunan Taiwan; ^2^ Division of Mental Health and Addiction Medicine National Health Research Institutes Zhunan Taiwan; ^3^ Graduate Program of Biotechnology in Medicine Department of Life Sciences NTHU & NHRI National Tsing Hua University Hsinchu Taiwan; ^4^ Ph.D. Program for Aging China Medical University Taichung Taiwan

**Keywords:** Alzheimer's disease, autophagy, non‐genomic vitamin D receptor, p53, vitamin D

## Abstract

Observational epidemiological studies have associated vitamin D deficiency with Alzheimer's disease (AD). However, whether vitamin D deficiency would result in some impacts on the vitamin D binding receptor (VDR) remains to be characterized in AD. Vitamin D helps maintain adult brain health genomically through binding with and activating a VDR/retinoid X receptor (RXR) transcriptional complex. Thus, we investigated the role of VDR in AD using postmortem human brains, APP/PS1 mice, and cell cultures. Intriguingly, although vitamin D was decreased in AD patients and mice, hippocampal VDR levels were inversely increased. The abnormally increased levels of VDR were found to be colocalized with Aβ plaques, gliosis and autophagosomes, implicating a non‐genomic activation of VDR in AD pathogenesis. Mechanistic investigation revealed that Aβ upregulated VDR without its canonical ligand vitamin D and switched its heterodimer binding‐partner from RXR to p53. The VDR/p53 complex localized mostly in the cytosol, increased neuronal autophagy and apoptosis. Chemically inhibiting p53 switched VDR back to RXR, reversing amyloidosis and cognitive impairment in AD mice. These results suggest a non‐genomic rewiring of VDR to p53 is key for the progression of AD, and thus VDR/p53 pathway might be targeted to treat people with AD.

## Graphical abstract

In Alzheimer’s brain, Aβ induces a non‐genomic action of vitamin D receptor (VDR) via switching VDR heterodimer partner from retinoid X receptor (RXR) to p53, in impairing autophagosome processing. Pharmacological inhibition of p53 switches VDR back to interaction with RXR and diminishes Aβ plaque load and cognitive deficits.

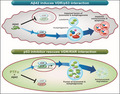

**Abbreviations**
AD: Alzheimer’s disease
ANOVA: analysis of variance
GSEA: gene set enrichment analysis
MPNC: mouse primary neural lineage cells
PBS: phosphate‐buffered saline
PFTα: pifithrin‐α
PLA: proximity ligation assay
qPCR: quantitative real‐time reverse transcription‐polymerase chain reaction
RXR: retinoid X receptor
SEM: standard error of the mean
VDR: vitamin D receptor
VDRE: vitamin D‐response element
WT: wild‐type

## 1 INTRODUCTION

Dementia is increasing in prevalence as the world's populations age (Masters et al., 2015). Alzheimer's disease (AD) accounts for 60%–80% of all cases of dementia (Alzheimer's Association, 2015; Kumar et al., 2015), but no therapy has been found to prevent or decelerate progression of this disease. Many epidemiological studies have suggested that vitamin D deficiency is linked to AD and other dementias (Tuohimaa, 2009). However, the mechanism underlying vitamin D‐associated pathogenesis of AD remains unclear. Vitamin D, in addition to its well‐known contribution to mineral and skeletal homeostasis, exerts neurotrophic or neuroprotective effects on the developing brain (Anastasiou et al., 2014). However, Vitamin D is not a vitamin but a steroid hormone (Demer et al., 2018). Like other steroid hormones, vitamin D may trigger both genomic and non‐genomic cellular responses. The genomic action of vitamin D is initiated via binding to vitamin D receptor (VDR). More specifically, the vitamin D metabolite 1α,25‐dihydroxyvitamin D3 (or calcitriol) binds to VDR. The ligand‐bound VDR prefers dimerization with retinoid X receptor (RXR) for transcription regulation of a set of genes containing vitamin D‐response elements (VDREs) in their promoter/regulatory regions. For instance, CYP24A1, a key vitamin D catabolism enzyme, is a typical VDRE‐containing target gene positively regulated by VDR (Ohyama et al., 1994; Zierold et al., 1995). There is much evidence that vitamin D exerts genomic actions in the brain and VDR has been found to be widely expressed among major brain cell types mediating brain development and function (Eyles et al., 2005).

Vitamin D also exerts non‐genomic actions on the brain, though less characterized (Hii & Ferrante, 2016; Zanatta et al., 2012). The non‐genomic action of VDR is a rapid plasma membrane response to cellular stimuli, but does not appear to require VDR–RXR interaction. VDR is also involved in xenobiotic metabolism (Krasowski et al., 2011; Reschly & Krasowski, 2006), independent of vitamin D binding (Li et al., 2007). Soluble toxic Aβ protofibrils are well‐known to cause neurodegeneration in AD (Suram et al., 2006). However, it remains unclear whether the non‐genomic VDR pathway functions in AD. So far most of the studies were focused on investigating the association of genetic variants of VDR with AD risk. There are some common VDR gene polymorphisms have been linked to the incidence of AD (Banerjee et al., 2015).

This study was prompted by epidemiological observation that vitamin D is usually deficient or low for many patients with AD and other dementia. However, we have been surprised that deficiencies in that vitamin have not been found to produce the same AD symptoms as has deficiencies in vitamin B12 or folate in humans (Landel et al., 2016; Smith & Refsum, 2016; Wang et al., 2001). Therefore, we began to wonder whether the genomic function of VDR could be impaired in AD. We performed a series of mechanistic investigations assessing expression and distribution of VDR in AD brains and sought to determine the role of VDR/p53 complex formation in the pathogenesis of AD with the use of a chemical inhibitor of p53 in AD mice. In all, this study provides support for a non‐genomic role of VDR pathway in promoting AD progression.

## 2 RESULTS

### 2.1 Increased brain VDR levels in human and mouse with AD

**FIGURE 1:**
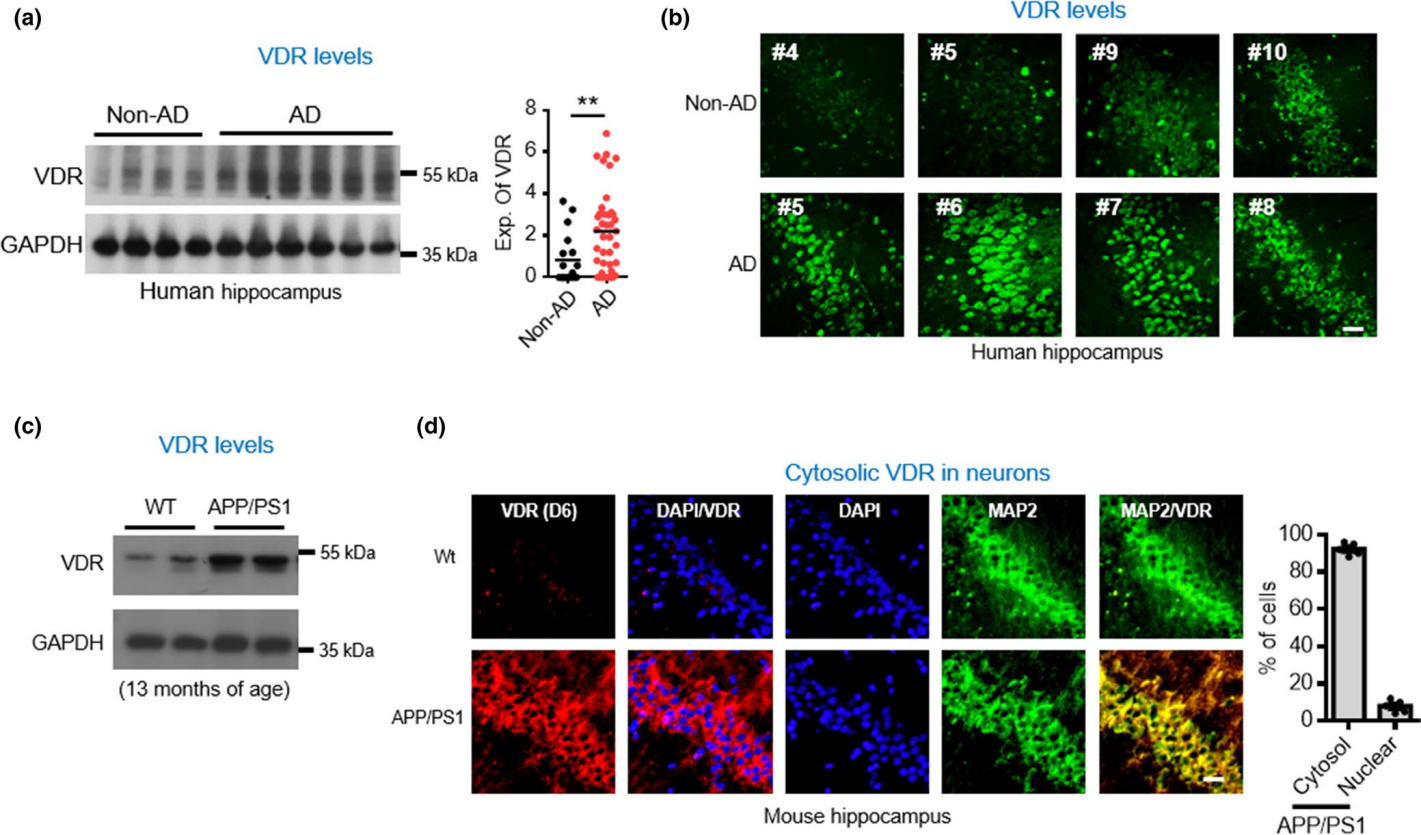
Increased VDR protein levels in hippocampal tissues of AD subjects and APP/PS1 mice. (a) Western blot analysis of VDR levels in hippocampus tissues of AD patients and age and gender‐matched non‐AD controls. Densitometrical quantification of the VDR level as ratios normalized with GAPDH (right panel, detail shown in Figure S1). **p < 0.01 by unpaired *t* test. (b) Immunohistochemistry analysis of VDR in sections of CA (Cornu Ammonis) regions in AD and non‐AD controls. Scale bars, 20 μm. (c) Western blot analysis of VDR levels in hippocampus of APP/PS1 and WT mice at 13 months of age. (d) Representative images of immunohistochemistry analysis for MAP2 (neuronal marker) and VDR in sections of CA regions of hippocampus in APP/PS1 mice. Right panel shows the quantification of the nuclear and cytoplasmic protein levels of VDR in AD brains (*n* = 5 mice). AD, Alzheimer’s disease; VDR, vitamin D receptor; WT, wild‐type

Since VDR functions as a ligand‐activated transcription factor upon vitamin D binding, we hypothesized that we might observe that VDR would be inhibited in response to deficiencies in vitamin D, its canonical ligand, in the brains of humans with AD. To find out, we performed Western blot and immunohistochemistry analyses on hippocampal tissues obtained postmortem from AD patients and found marked increases in VDR proteins (Figure 1a,b; Figure S1). We also found a similar phenomenon in APP/PS1 mouse brains. The older the mouse, the more evident the increase in VDR (Figure 1c; Figure S2a). In addition, this increase in VDR proteins was found in both humans and mice to be widely distributed among various cell types, including neurons, astrocytes and microglia (Figure 1d; Figure S2b). The increased VDR proteins were predominantly located in the cytosolic fraction (Figure 1d).

**FIGURE 2:**
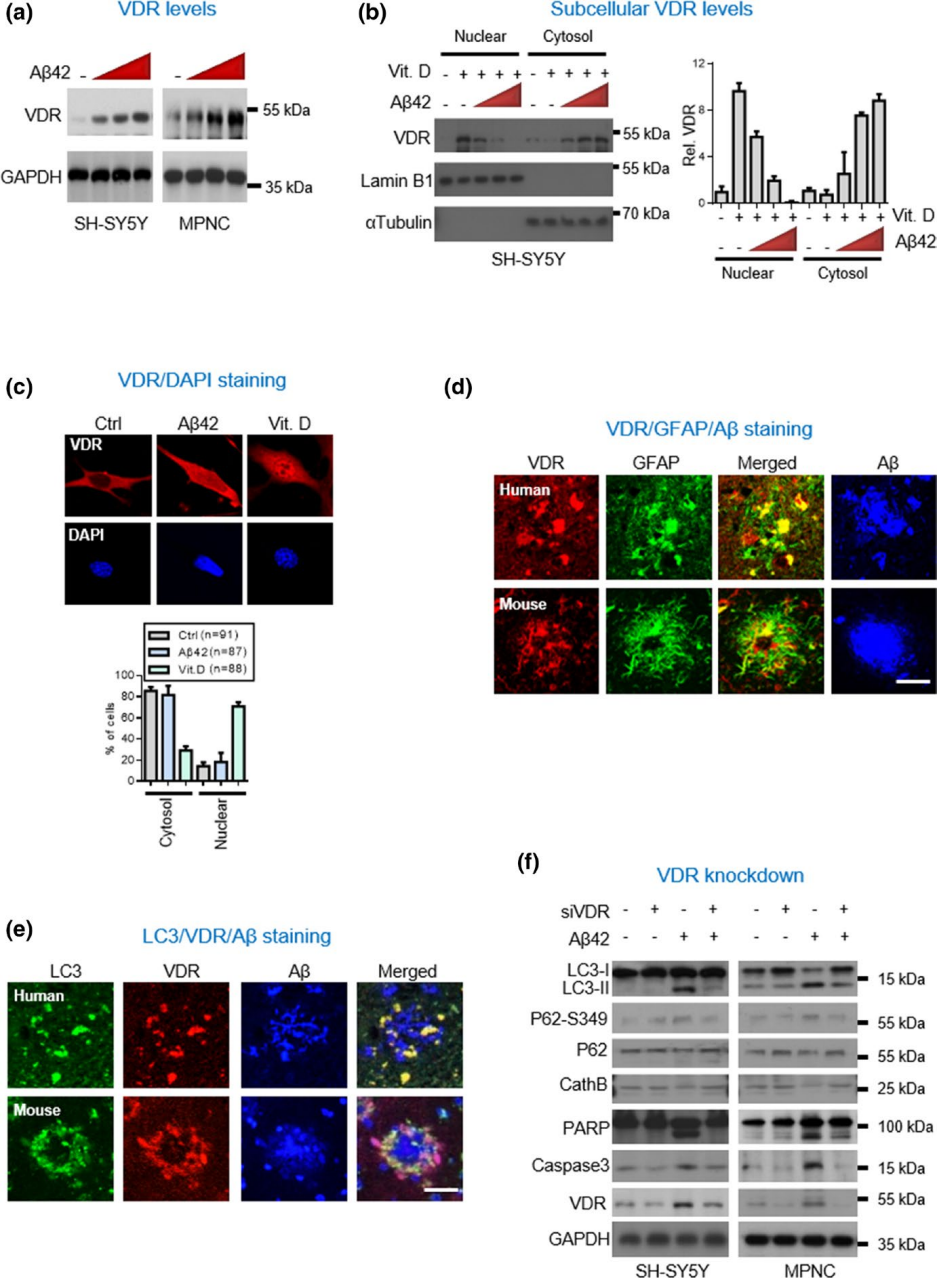
Cytosolic increase of VDR proteins in AD brains of human subjects and transgenic mice. (a) Dose‐dependent response of VDR protein expressions to Aβ42 treatment in neuronal cells. SH‐SY5Y and MPNC cells were treated with 0, 2, 4 or 6 μM Aβ42 for 6 h prior to western blot analysis of VDR protein levels. (b) Representative immunofluorescent staining shown β‐amyloid treatment increases predominantly cytosolic VDR protein level. Densitometrical quantification of the VDR level as ratios normalized with Lamin B or Tubulin. (c) VDR levels in cytoplasmic and nuclear fractions determined by Western blot analysis. SH‐SY5Y cells were treated with 100 nM calcitriol and 0, 2, 4 or 6 μM Aβ42 for 6 h prior to assays. The lower panel showed quantification results of the predominant nuclear and cytoplasmic localization of VDR. (d) Representative immunofluorescent micrographs of VDR/GFAP/Aβ triple staining in the hippocampal tissue sections of human and mouse AD brains. Scale bars, 20 μm. (e) Representative immunofluorescent micrographs of LC3/VDR/Aβ triple staining in the hippocampal tissue sections of human and mouse AD brains. Scale bars, 20 μm. (f) Western blot analysis of protein involved in apoptosis and autophagy in SH‐SY5Y and MPNC cells with or without treatment of Aβ42 and RNAi knockdown of VDR. The cells were pre‐treated with or without RNAi knockdown of VDR, and then treated with Aβ (6 μM) for 6 h before harvesting for western blot. AD, Alzheimer’s disease; MPNC, mouse primary neural lineage cells; VDR, vitamin D receptor

Because observed brain VDR protein levels appeared to us to be inversely upregulated in vitamin D deficient AD patients, we became interested in knowing whether this inverse relationship was a cellular response to the xenobiotic stimulus of Aβ, since VDR has been implicated in endobiotic/xenobiotic‐activated metabolism in a vitamin D‐independent manner (Krasowski et al., 2011). Adding increasing doses of Aβ42 to SH‐SY5Y cells, we found dose‐dependent increases in VDR protein levels in the absence of its canonical ligand, vitamin D, in these cells (Figure 2a). We next investigated whether these Aβ‐induced increases in VDR could be found in cytosol, since we had already found them in AD human and mouse brains. We conducted immunofluorescence staining of culture cells and prepared fractionated cytoplasmic and nuclear lysates for Western blot assay. Both assays clearly showed that the increased VDR proteins were largely retained in the cytosol when stimulated with Aβ42 (Figure 2b,c; Figure S3). The increase of cytosolic VDR in AD brains suggests a possible non‐genomic activity of VDR.

### 2.2 Abnormally increased VDR localization in autophagosomes, Aβ plaques and reactive gliosis

To find out how the increased VDR might be involved in the pathogenesis of AD at the cellular level, we first investigated whether the activated VDR might be associated with some of AD’s pathological features. We performed immunohistochemical staining of VDR in hippocampal tissues obtained from AD subjects and found pronounced increases in autophagy marker LC3, reactive gliosis, and Aβ plaques (Figure 2d,e; Figure S4). Since autophagy is a dysregulated process reportedly crucial in Aβ pathology (Di Meco et al., 2020), we explored how VDR might be involved in the pathogenesis of AD at the cellular level. We knocked down VDR in SH‐SY5Y cells and found abrogated Aβ‐induced autophagy and apoptosis (Figure 2f; Figure S5), indicating that VDR upregulation was a prerequisite to the induction of neuronal autophagy. Collectively, these studies of human brain sections, transgenic mice, and cell culture highlight the potential detrimental role that VDR may play in transducing Aβ neurotoxic signaling in AD.

### 2.3 Aβ induces disruption of VDR/RXR and formation of VDR/p53

**FIGURE 3:**
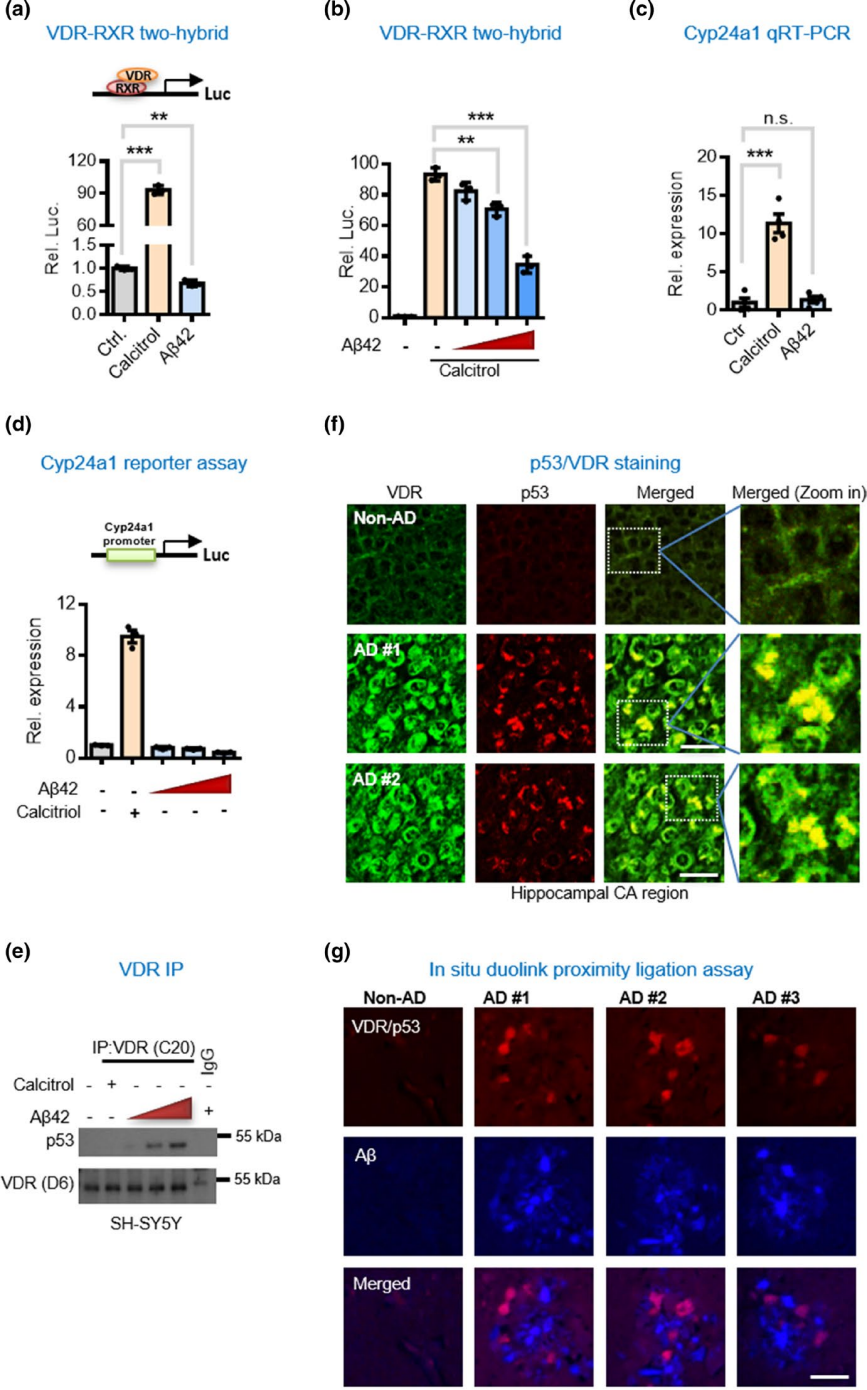
Aβ42 disrupts genomic VDR/RXR complex but induces the formation of VDR/p53 non‐genomic complex. (a) Mammalian two‐hybrid assays for studies of interaction of VDR with RXR in neuronal cells exposed to Aβ42. SH‐SY5Y cells were treated with 100 nM calcitriol or Aβ42 for 6 h before harvesting for mammalian two‐hybrid luciferase assays. Values are represented as the mean ± SEM and *p < 0.05, **p < 0.01 or ***p < 0.001 by unpaired *t* test. (b) Aβ42 decreases VDR–RXR interaction in a dose‐dependent manner. SH‐SY5Y cells were treated with 100 nM calcitriol and Aβ42 (2, 4 or 6 μM) for 6 h before harvesting for mammalian two‐hybrid luciferase assays. (c) Decreased Cyp24a1 (a known VDR target gene) gene expression in neuronal cells exposed to Aβ42. SH‐SY5Y cells were treated with 100 nM calcitriol and/or 4 μM Aβ42 for 6 h prior to qPCR analysis of the Cyp24a1 expressions. (d) Cyp24a1 promoter reporter assay in neuronal cells exposed to Aβ42. SH‐SY5Y cells were treated with 100 nM calcitriol or Aβ42 (2, 4 or 6 μM) for 6 h before harvesting for luciferase assays. (e) Western blot analysis of co‐immunoprecipitated VDR and p53 in neuronal cells. (f) Representative immunofluorescent micrographs showing p53 and VDR colocalized in hippocampal tissues of human AD brains. Hippocampal CA regions of AD patients were labelled with antibodies against VDR and p53. Scale bars, 20 μm. (g) Duolink^®^ proximity ligation assay for protein interaction between VDR and p53 in human hippocampal tissues. Scale bars, 20 μm. AD, Alzheimer’s disease; qPCR, quantitative real‐time reverse transcription‐polymerase chain reaction; RXR, retinoid X receptor; SEM, standard error of the mean; VDR, vitamin D receptor

We wanted to identify the potential molecular mechanisms underlying the non‐genomic activation and regulation of the VDR pathway in AD. In the genomic signaling pathway, vitamin D_3_ stimulates VDR to form a complex with RXR, which is then imported into the nucleus for transcription of many target genes (Orlov et al., 2012). However, our studies of hippocampal neurons of AD mice as well as in Aβ‐treated SH‐SY5Y cells showed that the enhanced signal of VDR to be largely retained in the cytoplasmic compartment and not translocated into the nucleus. Similarly, performing a biochemical assay, we did not find the formation in VDR/RXR heterodimer in Aβ‐treated SH‐SY5Y cells (Figure 3a), suggesting possible Aβ disablement of the VDR–RXR pathway. To make sure, we performed another biochemical study adding Aβ42 and vitamin D_3_ to the cell line above, and found the vitamin D_3_‐induced VDR/RXR interaction to be dose‐dependently abrogated by Aβ42 (Figure 3b). Moreover, the SH‐SY5Y cells treated with Aβ42 showed no induction of Cyp24a1, a canonical target gene of VDR, compared with a vitamin D_3_ positive control (Figure 3c). Finally, we directly assessed VDR transcriptional activity by reporter assay and found that the Aβ‐induced VDR protein did not induce target gene Cyp24a1 expression in SH‐SY5Y cells but vitamin D_3_ did (Figure 3d). Considered together, these results suggest that Aβ impairs the VDR–RXR pathway in AD.

**FIGURE 4:**
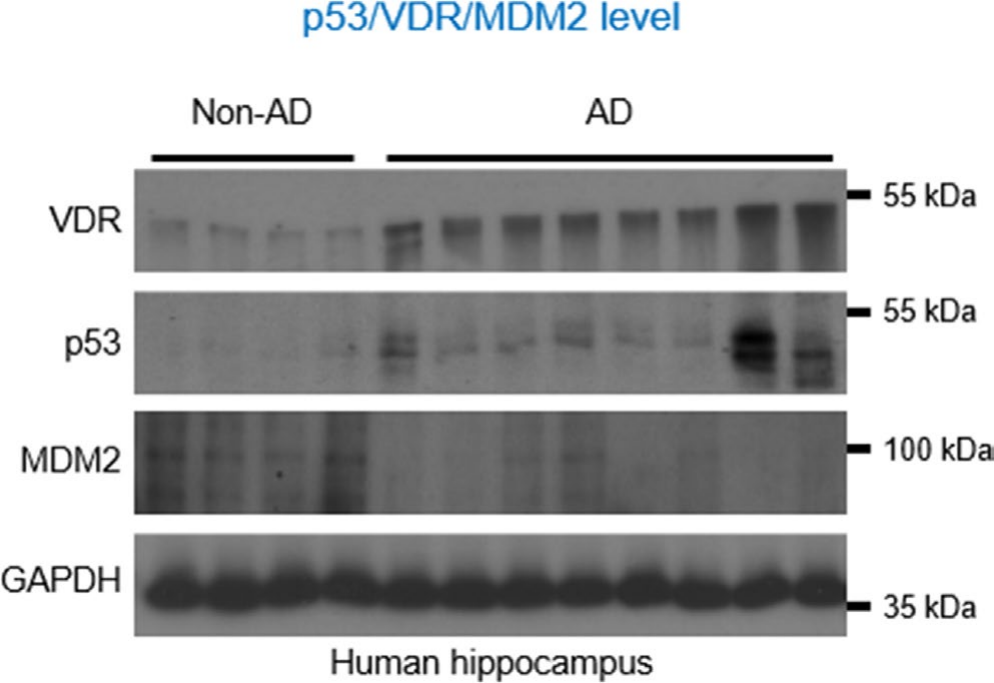
Concomitant upregulation of VDR and p53 proteins in the hippocampal tissues of AD. Western blot analysis of hippocampal tissue lysates from AD patients and normal controls with antibodies against p53, VDR, MDM2 and GAPDH. AD, Alzheimer’s disease; VDR, vitamin D receptor

Vitamin D receptor usually forms heterodimers with other transcription factors but we found no interaction between VDR and RXR, so we began to wonder whether VDR switched its interacting partnership with RXR to another transcription factor. P53 has been shown to interact with and modulate VDR activity in tumor cell apoptosis (Stambolsky et al., 2010), and most importantly, p53 protein has been found to be upregulated in AD brains (de la Monte et al., 1997). Therefore, we wanted to test whether VDR binding with RXR was rewired somehow to bind to p53. To determine whether the formation of VDR/p53 complex was induced by the direct cellular impact of Aβ42, we performed co‐immunoprecipitation assays finding that Aβ triggered VDR/p53 interaction in SH‐SY5Y cells and that this interaction was further enhanced with the addition of vitamin D3 (Figure 3e). Likewise, double‐labeling immunocytochemistry showed that both VDR and p53 were colocalized in the cytoplasm (Figure 3f). We performed a proximity ligation assay (PLA) to study the association between VDR and p53 in the postmortem brains of AD patients. As we had found in mice, VDR/p53 complex was abundant in the plaque regions in the brain sections (Figure 3g). Moreover, Western blot analysis also suggested that both VDR and p53 protein levels were increased while the levels of MDM2, an enzyme targeting p53 for degradation by ubiquitination, were concomitantly decreased in human brains (Figure 4). Consistent with the observations in AD brain tissues, the Western blot results also showed that both the VDR and p53 proteins were also predominantly localized within the cytosolic compartment in SH‐SY5Y cells exposed to Aβ42 (Figure S6). Considering these data together, we reasoned that the resulting VDR/p53 protein complex might exert non‐genomic actions on AD brains because the p53 and VDR proteins were found to be largely localized to the cytoplasm in AD hippocampal tissues and in SH‐SY5Y cells exposed to Aβ42.

### 2.4 Blocking p53 activity in AD mice reverses formation of VDR/RXR complex and ameliorates AD pathology

**FIGURE 5:**
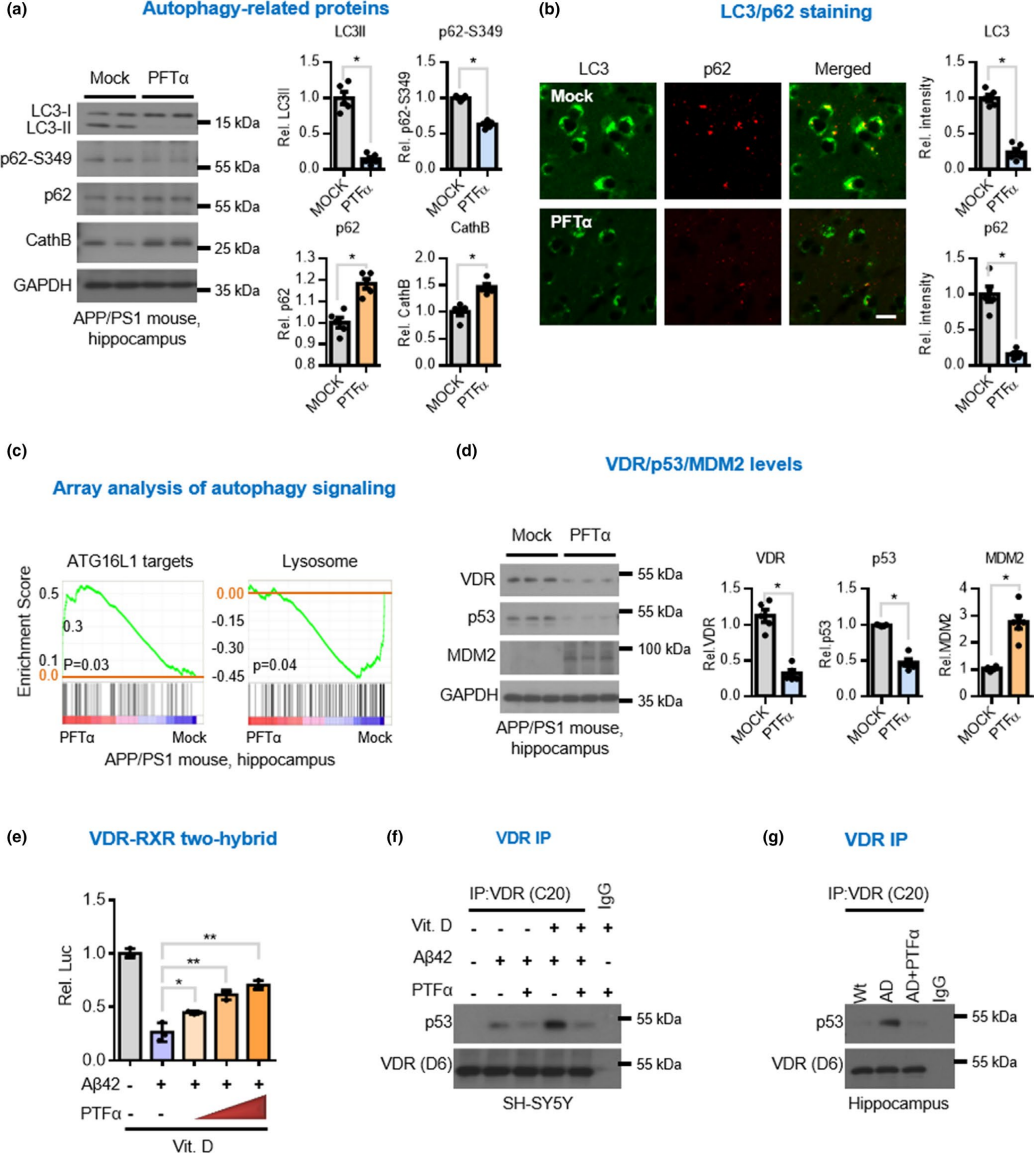
Inhibition of p53 ameliorates Aβ42‐induced autophagy and reverses interaction between VDR and RXR. (a) Western blot analysis of autophagic markers LC3, p62, ser349 phosphorylated p62 (p62‐S349), and lysosomal protease cathepsin B (cathB), in the hippocampal lysates of APP/PS1 mice. Four‐month‐old APP/PS1 mice raised on vitamin D_3_‐sufficient diets were intraperitoneally injected weekly with 3 mg/kg of p53 inhibitor PFTα for 8 months before harvesting hippocampal tissues for analysis. Densitometrical quantification of the protein level as ratios normalized with GAPDH (*n* = 5). (b) Representative immunofluorescent micrographs of LC3 and p62 staining in the hippocampal sections of APP/PS1 mice. Scale bars, 5 μm. The right panel showed quantification results of the protein level (*n* = 5). (c) GSEA showing significant functional gene sets differentially expressed in APP/PS1 mice after PFTα treatment, which include ATG16L1 (CADWELL_ATG16L1_TARGETS_UP) and lysosome (KEGG_LYSOSOME). The upregulation of ATG16L1 target genes refers to the reduction of ATG16L1 function. (d) Western blot analysis of VDR, p53 and MDM2 in the hippocampal lysates of APP/PS1 mice treated with or without p53 inhibitor. Densitometrical quantification of the protein level as ratios normalized with GAPDH (*n* = 5). (e) Mammalian two‐hybrid assays for studies of interaction of VDR with RXR in neuronal cells exposed to PFTα. (f, g) Western blot analysis of co‐immunoprecipitation of VDR/p53 complex in SH‐SY5Y cells (f) and in the hippocampus lysate of APP/PS1 mice (g) with the indicated treatments. Significantly different from control group at *p < 0.05 or **p < 0.01 by unpaired‐sample *t* test. AD, Alzheimer’s disease; GSEA, gene set enrichment analysis; PFTα, pifithrin‐α; RXR, retinoid X receptor; VDR, vitamin D receptor

Because both VDR and p53 are known to regulate autophagic activity (Jin, 2005; Sun, 2016; Wu & Sun, 2011) and the impaired autophagy flux is functionally linked to amyloid deposition and gliosis, we wanted to know whether the VDR/p53 complex might contribute to the impaired autophagic flux in AD. We performed an RNAi knockdown study of p53 and VDR in SH‐SY5Y cells and discovered that knocking down either p53 or VDR could reverse the autophagy and apoptosis caused by Aβ42 treatment alone or in combination with vitamin D_3_ (Figure S7), suggesting that the VDR/p53 interaction played an important role in mediating the Aβ‐induced autophagic neurodegeneration in AD. To validate this result *in vivo*, we treated AD mice with p53 inhibitor pifithrin‐α (PFTα) and found that this inhibition decreased their autophagic protein LC3II levels (Figure 5a,b). We also measured S349‐phosphorylated p62 (P‐S349) levels because site‐specific phosphorylation of p62 has been implicated in the disruption of autophagy‐mediated protein degradation in AD brains (Tanji et al., 2014). We found that p53 inhibitor decreased P‐S349 levels, which are normally increased in AD brain (Figure 5a, second panel). Lysosomal dysfunction has been found to lead to the accumulation of autophagosomes and neurodegeneration in AD (Zare‐Shahabadi et al., 2015). We also found that cathepsin B, a negative feedback regulator of lysosomal biogenesis (Qi et al., 2016), was concomitantly increased in brain tissues obtained from AD mouse brain (Figure 5a, 4th panel). Together, these findings further suggest that VDR/p53 plays an important role in impairing autophagic flux in AD and suggest that blocking the pathway could potentially restore the impairment in autophagy. To further our understanding, we conducted a microarray analysis to profile the gene expression response to PFTα treatment in AD mice. Like our Western blot and immunohistochemistry assays, gene set enrichment analysis (GSEA) revealed that treatment with the inhibitor enriched autophagy‐related genes in AD hippocampal tissues (Figure 5c). A tight coupling between autophagy and inflammatory stress in AD has previously been reported (Metaxakis et al., 2018; Zhong et al., 2016). Similarly, PFTα treatment effectively suppressed this inflammatory response (Figure S8). Considered together, these results suggest that the VDR‐p53 pathway may contribute to autophagic neurodegeneration in AD.

**FIGURE 6:**
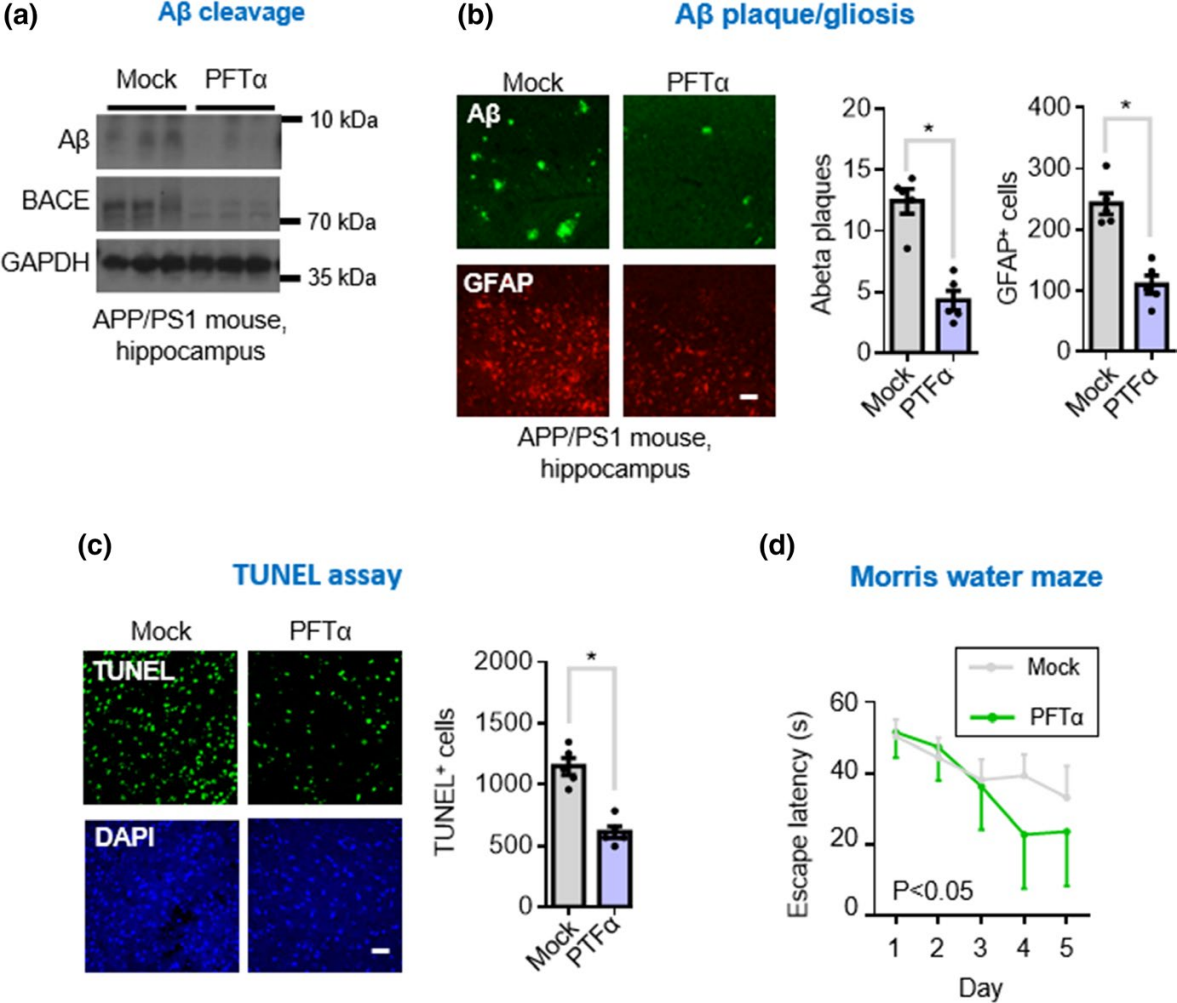
Reversal of brain pathology and cognitive function by inhibiting p53 activity in APP/PS1 AD mice. (a) Western blot analysis of Αβ and BACE (beta‐secretase enzyme) levels in the hippocampal lysates of APP/PS1 mice treated with or without p53 inhibitor. (b) Representative immunofluorescent micrographs of amyloid aggregates (anti‐Αβ) and gliosis (anti‐GFAP) double staining in the hippocampal tissue sections of APP/PS1 mice treated with or without p53 inhibitor (left panel). The average percentage of surface area with Αβ plaques and gliosis in five consecutive sections of hippocampus per animal (*n* = 5) was quantified by ImageJ and presented as the mean ± SEM (right panel). Significantly different from control group at *p < 0.05 by unpaired‐sample *t* test. Scale bars, 20 μm. (c) TUNEL immunofluorescent staining of brain sections in the APP/PS1 mice mentioned above. The TUNEL‐positive signals in five consecutive sections per animal (*n* = 5) were quantified by ImageJ and presented as the mean ± SEM (right panel). Significantly different from control group at *p < 0.05 by unpaired‐sample *t* test. Scale bars, 50 μm. (d) Cognitive performance assays for the AD mice treated with p53 inhibitor. APP/PS1 mice were given with or without weekly injections of PFTα (*n* = 6 mice) starting at the age of 4‐month. APP/PS1 mice at 12‐month of age were used for the Morris Water Maze test *p < 0.05 by ANOVA. AD, Alzheimer’s disease; ANOVA, analysis of variance; PFTα, pifithrin‐α; SEM, standard error of the mean

Finally, since the impaired autophagic signaling was effectively rescued by the p53 inhibition, we performed an experiment to determine whether the molecular and morphological changes that occur in AD brain as well as AD‐related cognitive decline could also be ameliorated with the use of p53 inhibitor. The inhibitor not only decreased protein levels of p53 and VDR (Figure 5d) but it also concomitantly increased the levels of MDM2 (Figure 3, 5, 3rd panel), both with or without vitamin D_3_ supplementation. It should also be noted that the disrupted VDR/RXR interaction was also found to be restored (Figure 5e) following p53 inhibitor treatment (Figure 5f,g), suggesting that disrupting the VDR/p53 complex could reverse the binding of VDR to RXR. Based on these findings, we believed we would also be able to observe improvement in brain lesions. We performed Western blot and immunohistochemistry studies of AD brain tissues and found significant attenuation in Aβ deposits, BACE (beta‐secretase enzyme) activity, reactive gliosis, and neuronal apoptosis, regardless of vitamin D_3_ supplementation (Figure 6a–c). Finally, we wanted to know whether cognitive functioning and performance behavior would also be improved by treatment with p53 inhibitor. Mice administered intraperitoneally the p53 inhibitor showed significant improvement in both the Morris Water Maze test (Figure 6d). These results suggest that VDR‐p53 pathway might be targeted therapeutically in the treatment of AD.

## 3 DISCUSSION

This study demonstrated how VDR could be activated by a non‐genomic mechanism independent of classical vitamin D ligand. This unique signaling pathway underlies the importance of VDR/p53 pathway in activating autophagic apoptosis in AD.

By exploring the mechanism of non‐genomic VDR activation, we were able to demonstrate that the genomic VDR–RXR signaling pathway to be compromised by Aβ in AD, leaving the VDR/p53 complex to form in the cytosol and cause damage to AD brains. To determine whether the non‐genomic activation of VDR/p53 played a role in AD, we used a chemical inhibitor of p53 to block the negative activity of VDR/p53 and reverse the brain pathology and cognition impairment in the AD mouse model. This suggests that the VDR/p53 signaling could potentially be used as a therapeutic target in the treatment of AD. However, it should be noted that use of p53 inhibitor could potentially increase the risk of tumor development because it is a key tumor suppressor protein. Consequently, further studies are needed to identify upstream modulators or downstream effectors for VDR‐p53 signaling to avoid potential pitfalls.

Although it might be a surprise to find an inverse correlation of VDR protein levels and the concentration of its canonical ligand vitamin D in serum, other studies also report similar results that may support this finding. Some studies have found older African‐Americans are two to three times more likely to develop AD than elderly caucasians (Alzheimer's, 2014; Amadori et al., 2017) and studies have reported that although African‐American have higher mean VDR levels (Amadori et al., 2017; O'Neill et al., 2013; Richards et al., 2017), they have much lower serum vitamin D concentrations (Dawson‐Hughes, 2004). Another example of the converse relationship between vitamin D concentration and VDR levels has been reported in patients with insulin resistance and obesity, who have been found have deficient levels of vitamin D on the one hand but increased levels of VDR in adipose tissue on the other (Kang et al., 2015). Notably, in addition to AD, patients with vascular disease, thyroid disorders, and osteoporosis are most likely to have decreased levels of serum vitamin D and, of course, be at higher risk for dementia (Autier et al., 2014; Duthie et al., 2011). Therefore, future studies may want to explore whether the decrease of vitamin D levels may be an early disease manifestation in these diseases and not a common cause.

Vitamin D deficiency in early childhood has been linked to impaired neurodevelopment and skeletal health, and the most effective and accepted approach to resolving this issue has been through vitamin D supplementation (Society for Adolescent Health & Medicine, 2013). The same deficiency in seniors is also considered as a common health risk factor that affects dementia, cardiovascular diseases, diabetes, cancers and several other chronic illnesses and geriatric syndromes. However, the need for its supplementation to prevent these diseases is currently debatable (Grant & Boucher, 2020; Lucas & Wolf, 2019). If the genomic pathway of VDR has been compromised, then it might be doubtful whether vitamin D repletion strategy could protect against AD. In fact, several recent randomized clinical trials have demonstrated that correcting vitamin D deficiencies does not have any genuine health benefits in the reduction of cardiovascular disease, type 2 diabetes, or chronic kidney disease (Lucas & Wolf, 2019). Therefore, older adults with dementia might want to exercise some caution when deciding to take or continue using vitamin D supplements.

## 4 METHODS

### 4.1 Mice

Double transgenic APP/PS1 mice (Cat# 037565‐JAX, RRID:MMRRC_037565‐JAX) were purchased from Jackson Laboratory to breed with wild‐type B6C3F1/Bltw (C57BL/6N background) mice. Mice were weaned at 4‐weeks of age (±3 days) and fed with the subnormal dosage of vitamin D_3_ diet (600 IU/kg of cholecalciferol, corresponding to an intake of 0.06 mcg/day). Since it has been reported that feeding mice with 1–2 mcg of cholecalciferol per day for 12 weeks can significantly increase serum 25(OH)D_3_ but do not influence serum calcium level, APP/PS1 mice at 4 months of age were divided randomly into experimental group and control group. The mice of the experimental group were fed with 0.8 mcg of cholecalciferol per day (as D_3_‐supplemented diet; Research Diets, Inc; Match Altromin 1320 with 8044 IU Vitamin D3/kg; Cat# D13031002) and control groups with 0.06 mcg per day (control diet; Altromin 1320 diet; Cat# Altromin 1320) for 2–8 months before assays. To block p53 activation in AD, the four‐month‐old APP/PS1 mice under vitamin D_3_‐sufficient diet condition were intraperitoneally injected weekly with 3 mg/kg of p53 inhibitor PFTα (Sigma‐Aldrich; Cat# P4359) for 3 months before harvesting hippocampal tissues for analysis. For the Morris water maze test and nest construction behavior assays, PFTα was given to AD mice for 8 months. Serum 25(OH)D_3_ levels in APP/PS1 and wild‐type mice were determined by Vitamin D_3_ EIA Kit (Cayman Chemical; Cat# 501050) at the indicated time points. All experimental animal procedures and protocols were approved by the Institutional Animal Care and Use Committee at NHRI (approved protocol no. NHRI‐IACUC‐101057‐A and NHRI‐IACUC‐103136‐A).

### 4.2 Human brain tissue and ethics statement

All human brain tissues were obtained from the Brain and Tissue Bank at the University of Maryland. In total, 58 brain tissues were used. Among them, 40 were from individuals with a clinical diagnosis of probable AD, which includes 21 men and 19 women, with an average age of 80.1 ± 8.8 years, and postmortem interval of 10.25 ± 6.7 h. Another 18 brain tissues were from individuals without neurological disorders, which include 11 men and 7 women, with an average age of 74.1 ± 6.9 years, and a postmortem interval of 14.9 ± 8.6 h. All studies and protocols were approved by the Research Ethics Committee at National Health Research Institutes (approved protocol no. EC1001103).

### 4.3 Antibody

The antibodies used in this study are listed as follows: VDR (C20), Santa Cruz Biotechnology, Cat# sc‐1008, RRID:AB_632070; VDR(D6), Santa Cruz, Biotechnology, Cat# sc‐13133, RRID:AB_628040; GAPDH, GeneTex, Cat# GTX100118, RRID:AB_1080976; Tubulin, GeneTex, Cat# GTX112141, RRID:AB_10722892; PARP‐1/2 (H‐250), Santa Cruz Biotechnology, Cat# sc‐7150, RRID:AB_216073; LC3B, Cell Signaling Technology, Cat# 4108, RRID:AB_2137703; Beta‐Amyloid‐1–16 antibody, BioLegend, Cat# 803014, RRID:AB_2728527; β‐Amyloid Antibody, Cell Signaling Technology, Cat# 2454, RRID:AB_2056585; BACE (M‐83), Santa Cruz Biotechnology, Cat# sc‐10748, RRID:AB_2061505; GFAP (Clone SP78), MybioSourse, Cat# MBS302899, DISCONTINUED; GFAP (GA5), Cell Signaling Technology, Cat# 3670, RRID:AB_561049; p53 (DO‐1)Santa Cruz Biotechnology Cat# sc‐126, RRID:AB_628082; Cathepsin B Antibody (FL‐339), Santa Cruz Biotechnology, Cat# sc‐13985, RRID:AB_2261223; Phospho‐SQSTM1/p62 (Ser349), Cell Signaling Technology, Cat# 95697, RRID:AB_2800251; SQATM1/p62 (GT1478), Thermo Fisher Scientific, Cat# MA5‐27800, RRID:AB_2735371; TNF‐α (D2D4) XP^®^ Rabbit mAb, Cell Signaling Technology, Cat# 11948, RRID:AB_2687962; Lamin B (M‐20) antibody, Santa Cruz Biotechnology, Cat# sc‐6217, RRID:AB_648158; Alexa 488 chicken anti‐rabbit IgG(H+L), Thermo Fisher Scientific, Cat# A‐21441, RRID:AB_2535859; Alexa 594 chicken anti‐mouse IgG(H+L), Thermo Fisher Scientific, at# A‐21201, RRID:AB_2535787; Alexa 594 chicken anti‐rabbit IgG(H+L), Thermo Fisher Scientific, Cat# A‐21442, RRID:AB_2535860; Alexa 488 chicken anti‐goat IgG(H+L), Thermo Fisher ScientificCat# A‐21468, RRID:AB_2535871; Peroxidase‐AffiniPure Goat Anti‐Rabbit IgG (H+L), Jackson ImmunoResearch Labs, Cat# 111–035–144, RRID:AB_2307391; Peroxidase‐AffiniPure Goat Anti‐Mouse IgG (H+L), Jackson ImmunoResearch Labs, Cat# 115–035–146, RRID:AB_2307392; Peroxidase‐AffiniPure Rabbit Anti‐Goat IgG (H+L), Jackson ImmunoResearch Labs, Cat# 305–035–003, RRID:AB_2339400; Mouse anti‐Rabbit light chain; HRP conjugate, Millipore, at# MAB201P, RRID:AB_827270; HRP‐conjugated AffiniPure Mouse Anti‐Rabbit IgG Light Chain, Bclonal Cat# AS061, RRID:AB_2864055; HRP‐conjugated AffiniPure Goat Anti‐Mouse IgG Light Chain antibody, ABclonal, Cat# AS062, RRID:AB_2864056; Duolink In Situ PLA Probe Anti‐Rabbit PLUS antibody, Sigma‐Aldrich, Cat# DUO92002, RRID:AB_2810940; Duolink In Situ PLA Probe Anti‐Mouse MINUS Antibody, Sigma‐Aldrich, Cat# DUO92004, RRID:AB_2713942.

### 4.4 Cell culture

The following cell lines were used in this study: SH‐SY5Y (human neuroblastoma, ATCC CRL‐2266), IMR‐32 (human neuroblastoma, ATCC CCL‐127) and MPNC (mouse primary neural lineage cells, a gift from Dr. Hsing I Huang at Chang Gung University). SH‐SY5Y and IMR32 were cultured in MEM (minimun essential media) (Invitrogen) and MPNC in Dulbecco’s modified Eagle’s medium (Invitrogen). Cells were grown at 37°C in a 5% CO_2_ humid atmosphere.

### 4.5 Cells transfection, mammalian two‐hybrid assay and Cyp24a1 reporter assay

Oligomeric β‐amyloid (Aβ42; Sigma‐Aldrich) was prepared as described previously (Stine et al. 2011). RNAi‐mediated knockdown of VDR or p53 was performed by transient transfection of siVDR, sip53, or a control siRNA (Stealth siRNA; Invitrogen) into SH‐SY5Y cells with DharmaFECT (Dharmacon) at a concentration of 50 nM in 6‐well culture plates. Transient overexpression of VDR in SH‐SY5Y cells was performed by transfecting a VDR expression plasmid (pcDNA3‐VDR) or a mock control plasmid (pcDNA3) with Lipofectamine 2000 (Invitrogen). For mammalian two‐hybrid assays to assess the interaction between VDR and RXR, SH‐SY5Y cells were co‐transfected with 100 ng of pCMV‐BD‐RXRα (as bait), 100 ng of pCMV‐AD‐VDR (as prey), 500 ng of pFR‐luc (as a reporter) and a control plasmid (pRL‐null constitutively expressing low levels of Renilla reniformis) in 6‐well plates as described in Dr. Jurutka's publication (Bartik et al., 2010) with minor modifications. The transfected cells were then treated with Aβ42 (1–4 μM) or calcitriol (100 nM) for 6 h before luciferase activity assay (Dual‐Luciferase Reporter Assay, Promega). A 587‐bp region of the human Cyp24a1 gene promoter that contains two VDRE motifs was cloned into the promoter‐less luciferase expression vector pGL3‐basic (Promega) (Luo et al., 2010). The transfected cells were then treated with Aβ42 (1–4 μM) or calcitriol (100 nM) for 6 h before Dual‐Luciferase Reporter Assay.

### 4.6 In situ PLA

A Duolink^®^ PLA Starter Kits ((Sigma‐Aldrich) was used to detect in situ PLA for VDR/p53 interactions in postmortem brain tissues. Paraffin‐embedded human brain sections were incubated with mouse anti‐p53 (DO‐1) and rabbit anti‐VDR (C20) antibodies (Santa Cruz Biotech) in antibody diluent buffer overnight at 4°C, followed by incubation with Duolink anti‐mouse MINUS and anti‐ Rabbit PLUS secondary antibodies for 1 h. For detection, the Duolink in situ detection reagent‐RED was used.

### 4.7 Cell viability and TUNEL staining

Colorimetric WST‐1 assay (Roche) was used to determine cell viability. The absorbance was measured by a spectrophotometer (SpectraMax Plus from Molecular Devices) at 450 nm against a reference at 690 nm. The optical density values relative to the control cells in the assay represent the percentage of viable cells. To detect cell apoptosis, the TUNEL assay was performed using the ApoAlert™ DNA Fragmentation Assay Kit (Clontech) to detect the presence of DNA fragmentation in frozen tissue sections. The fixed sections were washed twice with phosphate‐buffered saline (PBS) before incubating in the permeabilization solution (0.2% Triton X‐100 in PBS) on ice for 10 min. The sections were washed twice in PBS and then incubated in TUNEL reaction mixture at 37°C in the dark in a humidified atmosphere for 1 h. The stained sections were washed once again with PBS before mounting with 4′,6‐diamidino‐2‐phenylindole mounting medium (VECTASHIELD) for fluorescence microscopy analysis.

### 4.8 Quantitative real‐time reverse transcription‐polymerase chain reaction

Total RNA from brain tissues or culture cells were extracted using the illustra RNAspin Mini RNA Isolation Kit (GE Healthcare Life Sciences) for reverse transcription with the High‐Capacity cDNA Reverse Transcription Kits (ABI Applied Biosystems) according to the manufacturer's instructions. The quantitative real‐time Reverse Transcription‐PCR analysis was performed using the Fast SYBR Green Master Mix (ABI Applied Biosystems). Results were determined using respective standard curves calculations. The primers used in this study are listed as follows: Human VDR‐F: 5′‐CGA CCC CAC CTA CTC CGA CTT‐3′; Human VDR‐R 5′‐GGC TCC CTC CAC CAT CAT TC‐3′; Mouse VDR‐F: 5′‐GGA GCT ATT CTC CAA GGC CC‐3′; Mouse VDR‐R: 5′‐GGG TCA TCG GAG CCT TCT TC‐3′; Human GAPDH‐F: 5′‐CCT GCC AAA TAT GAT GAC ATC AAG‐3′; Human GAPDH‐R: 5′‐ACC CTG TTG CTG TAG CCA AA‐3′; Mouse GAPDH‐F: 5′‐AAG GTC ATC CCA GAG CTG AA‐3′; Mouse GAPDH‐R: 5′‐CTG CTT CAC CAC CTT CTT GA‐3′; Human Cyp24a1‐F: 5′‐CAT CAT GGC CAT CAA AAC AAT‐3′; Human Cyp24a1: 5′‐GCA GCT CGA CTG GAG TGA C‐3′.

### 4.9 Microarray and GSEA gene sets analysis

The microarray of mouse Clariom S Assays (Thermo Fisher Scientific) for whole‐transcript expression analysis was used in this study. We compared the gene expression profiles of hippocampal tissues from the APP/PS1 mice treated with or without p53 inhibitor (PFTα, 3 mg/kg). To analyze the signaling pathways that were impacted by the p53 inhibitor, we performed GSEA for the C2‐curated gene sets and H‐hallmark gene sets with a size of >15 genes. *p* < 0.05 was considered significant.

### 4.10 Quantification and statistical analysis

Statistical information, including *n* (number of patients or mice), mean and statistical significance values, is indicated in the figure legends. None specific method was used to determine whether the data met assumptions of the statistical approach. Statistical significance was determined with Graphpad Prism 6 using the tests indicated in each figure. Data were considered statistically significant at *p* < 0.05.

## CONFLICT OF INTEREST

The authors declare no competing interests.

## AUTHOR CONTRIBUTIONS

**Jyh‐Lyh Juang, Rai‐Hua Lai, Yueh‐Ying Hsu:** Conceptualization. **Rai‐Hua Lai, Yueh‐Ying Hsu, Che‐Ching Huang, Jyh‐Lyh Juang, Mei‐Hsin Chen, Feng‐Shiun Shie:** Methodology. **Rai‐Hua Lai, Yueh‐Ying Hsu, Jyh‐Lyh Juang, Mei‐Hsin Chen, Feng‐Shiun Shie:** Investigation. **Rai‐Hua Lai, Yueh‐Ying Hsu, Jyh‐Lyh Juang, Mei‐Hsin Chen**: Visualization. **Jyh‐Lyh Juang:** Supervision. **Jyh‐Lyh Juang, Rai‐Hua Lai:** Writing – original draft. **Jyh‐Lyh Juang:** Writing – review & editing.

## Supporting information

Fig S1‐S8

## Data Availability

The data that support the findings of this study are available from the corresponding author upon reasonable request.

## References

[acel13509-bib-0001] Alzheimer's Association . (2014). 2014 Alzheimer's disease facts and figures. Alzheimer's & Dementia: The Journal of the Alzheimer's Association, 10(2), e47–e92. 10.1016/j.jalz.2014.02.001 24818261

[acel13509-bib-0002] Alzheimer's Association . (2015). 2015 Alzheimer's disease facts and figures. Alzheimer's & Dementia: The Journal of the Alzheimer's Association, 11(3), 332–384. 10.1016/j.jalz.2015.02.003 25984581

[acel13509-bib-0003] Amadori, D. , Serra, P. , Masalu, N. , Pangan, A. , Scarpi, E. , Maria Bugingo, A. , Katabalo, D. , Ibrahim, T. , Bongiovanni, A. , Miserocchi, G. , Spadazzi, C. , Liverani, C. , Turri, V. , Tedaldi, R. , & Mercatali, L. (2017). Vitamin D receptor polymorphisms or serum levels as key drivers of breast cancer development? The question of the vitamin D pathway. Oncotarget, 8(8), 13142–13156. 10.18632/oncotarget.14482 28061456 PMC5355083

[acel13509-bib-0004] Anastasiou, C. A. , Yannakoulia, M. , & Scarmeas, N. (2014). Vitamin D and cognition: An update of the current evidence. Journal of Alzheimer's Disease, 42(Suppl 3), S71–S80. 10.3233/JAD-132636 24820017

[acel13509-bib-0005] Autier, P. , Boniol, M. , Pizot, C. , & Mullie, P. (2014). Vitamin D status and ill health: A systematic review. The Lancet Diabetes & Endocrinology, 2(1), 76–89. 10.1016/S2213-8587(13)70165-7 24622671

[acel13509-bib-0006] Banerjee, A. , Khemka, V. K. , Ganguly, A. , Roy, D. , Ganguly, U. , & Chakrabarti, S. (2015). Vitamin D and Alzheimer's disease: Neurocognition to therapeutics. International Journal of Alzheimer's Disease, 2015, 1–11. 10.1155/2015/192747 PMC4553343 26351614

[acel13509-bib-0007] Bartik, L. , Whitfield, G. K. , Kaczmarska, M. , Lowmiller, C. L. , Moffet, E. W. , Furmick, J. K. , Hernandez, Z. , Haussler, C. A. , Haussler, M. R. , & Jurutka, P. W. (2010). Curcumin: A novel nutritionally derived ligand of the vitamin D receptor with implications for colon cancer chemoprevention. Journal of Nutritional Biochemistry, 21(12), 1153–1161. 10.1016/j.jnutbio.2009.09.012 PMC2891903 20153625

[acel13509-bib-0008] Dawson‐Hughes, B. (2004). Racial/ethnic considerations in making recommendations for vitamin D for adult and elderly men and women. American Journal of Clinical Nutrition, 80(6 Suppl), 1763S–1766S. 10.1093/ajcn/80.6.1763S 15585802

[acel13509-bib-0009] de la Monte, S. M. , Sohn, Y. K. , & Wands, J. R. (1997). Correlates of p53‐ and Fas (CD95)‐mediated apoptosis in Alzheimer's disease. Journal of the Neurological Sciences, 152(1), 73–83. 10.1016/S0022-510X(97)00131-7 9395128

[acel13509-bib-0010] Demer, L. L. , Hsu, J. J. , & Tintut, Y. (2018). Steroid hormone vitamin D: Implications for cardiovascular disease. Circulation Research, 122(11), 1576–1585. 10.1161/CIRCRESAHA.118.311585 29798901 PMC6122607

[acel13509-bib-0011] Di Meco, A. , Curtis, M. E. , Lauretti, E. , & Pratico, D. (2020). Autophagy dysfunction in Alzheimer's disease: Mechanistic insights and new therapeutic opportunities. Biological Psychiatry, 87(9), 797–807. 10.1016/j.biopsych.2019.05.008 31262433

[acel13509-bib-0012] Duthie, A. , Chew, D. , & Soiza, R. L. (2011). Non‐psychiatric comorbidity associated with Alzheimer's disease. QJM, 104(11), 913–920. 10.1093/qjmed/hcr118 21768167

[acel13509-bib-0013] Eyles, D. W. , Smith, S. , Kinobe, R. , Hewison, M. , & McGrath, J. J. (2005). Distribution of the vitamin D receptor and 1 alpha‐hydroxylase in human brain. Journal of Chemical Neuroanatomy, 29(1), 21–30. 10.1016/j.jchemneu.2004.08.006 15589699

[acel13509-bib-0014] Grant, W. B. , & Boucher, B. J. (2020). Health outcomes with vitamin D supplementation. JAMA, 323(16), 1618–1619. 10.1001/jama.2020.2642 32343324

[acel13509-bib-0015] Hii, C. S. , & Ferrante, A. (2016). The non‐genomic actions of vitamin D. Nutrients, 8(3), 135. 10.3390/nu8030135 26950144 PMC4808864

[acel13509-bib-0016] Jin, S. (2005). p53, autophagy and tumor suppression. Autophagy, 1(3), 171–173. 10.4161/auto.1.3.2051 16874039

[acel13509-bib-0017] Kang, S. , Tsai, L. T. , Zhou, Y. , Evertts, A. , Xu, S. U. , Griffin, M. J. , Issner, R. , Whitton, H. J. , Garcia, B. A. , Epstein, C. B. , Mikkelsen, T. S. , & Rosen, E. D. (2015). Identification of nuclear hormone receptor pathways causing insulin resistance by transcriptional and epigenomic analysis. Nature Cell Biology, 17(1), 44–56. 10.1038/ncb3080 25503565 PMC4281178

[acel13509-bib-0018] Krasowski, M. D. , Ni, A. , Hagey, L. R. , & Ekins, S. (2011). Evolution of promiscuous nuclear hormone receptors: LXR, FXR, VDR, PXR, and CAR. Molecular and Cellular Endocrinology, 334(1–2), 39–48. 10.1016/j.mce.2010.06.016 20615451 PMC3033471

[acel13509-bib-0019] Kumar, A. , Singh, A. , & Ekavali. (2015). A review on Alzheimer's disease pathophysiology and its management: An update. Pharmacological Reports, 67(2), 195–203. 10.1016/j.pharep.2014.09.004 25712639

[acel13509-bib-0020] Landel, V. , Annweiler, C. , Millet, P. , Morello, M. , & Feron, F. (2016). Vitamin D, cognition and Alzheimer's disease: The therapeutic benefit is in the D‐tails. Journal of Alzheimer's Disease, 53(2), 419–444. 10.3233/JAD-150943 PMC4969697 27176073

[acel13509-bib-0021] Li, Q. P. , Qi, X. , Pramanik, R. , Pohl, N. M. , Loesch, M. , & Chen, G. (2007). Stress‐induced c‐Jun‐dependent vitamin D receptor (VDR) activation dissects the non‐classical VDR pathway from the classical VDR activity. Journal of Biological Chemistry, 282(3), 1544–1551. 10.1074/jbc.M604052200 17121851

[acel13509-bib-0022] Lucas, A. , & Wolf, M. (2019). Vitamin D and health outcomes: Then came the randomized clinical trials. JAMA, 322, 1866. 10.1001/jama.2019.17302 31703117

[acel13509-bib-0023] Luo, W. , Karpf, A. R. , Deeb, K. K. , Muindi, J. R. , Morrison, C. D. , Johnson, C. S. , & Trump, D. L. (2010). Epigenetic regulation of vitamin D 24‐hydroxylase/CYP24A1 in human prostate cancer. Cancer Research, 70(14), 5953–5962. 10.1158/0008-5472.CAN-10-0617 20587525 PMC2928678

[acel13509-bib-0024] Masters, C. L. , Bateman, R. , Blennow, K. , Rowe, C. C. , Sperling, R. A. , & Cummings, J. L. (2015). Alzheimer's disease. Nature Reviews Disease Primers, 1, 15056. 10.1038/nrdp.2015.56 27188934

[acel13509-bib-0025] Metaxakis, A. , Ploumi, C. , & Tavernarakis, N. (2018). Autophagy in age‐associated neurodegeneration. Cells, 7(5). 37, 10.3390/cells7050037 PMC5981261 29734735

[acel13509-bib-0026] O'Neill, V. , Asani, F. F. , Jeffery, T. J. , Saccone, D. S. , & Bornman, L. (2013). Vitamin D receptor gene expression and function in a South African population: Ethnicity, vitamin D and FokI. PLoS One, 8(6), e67663. 10.1371/journal.pone.0067663 23805323 PMC3689684

[acel13509-bib-0027] Ohyama, Y. , Ozono, K. , Uchida, M. , Shinki, T. , Kato, S. , Suda, T. , Yamamoto, O. , Noshiro, M. , & Kato, Y. (1994). Identification of a vitamin D‐responsive element in the 5'‐flanking region of the rat 25‐hydroxyvitamin D3 24‐hydroxylase gene. Journal of Biological Chemistry, 269(14), 10545–10550. 10.1016/S0021-9258(17)34094-2 8144641

[acel13509-bib-0028] Orlov, I. , Rochel, N. , Moras, D. , & Klaholz, B. P. (2012). Structure of the full human RXR/VDR nuclear receptor heterodimer complex with its DR3 target DNA. EMBO Journal, 31(2), 291–300. 10.1038/emboj.2011.445 PMC3261568 22179700

[acel13509-bib-0029] Qi, X. , Man, S. M. , Malireddi, R. K. S. , Karki, R. , Lupfer, C. , Gurung, P. , Neale, G. , Guy, C. S. , Lamkanfi, M. , & Kanneganti, T.‐D. (2016). Cathepsin B modulates lysosomal biogenesis and host defense against Francisella novicida infection. Journal of Experimental Medicine, 213(10), 2081–2097. 10.1084/jem.20151938 PMC5030800 27551156

[acel13509-bib-0030] Reschly, E. J. , & Krasowski, M. D. (2006). Evolution and function of the NR1I nuclear hormone receptor subfamily (VDR, PXR, and CAR) with respect to metabolism of xenobiotics and endogenous compounds. Current Drug Metabolism, 7(4), 349–365. 16724925 10.2174/138920006776873526 PMC2231810

[acel13509-bib-0031] Richards, Z. , Batai, K. , Farhat, R. , Shah, E. , Makowski, A. , Gann, P. H. , Kittles, R. , & Nonn, L. (2017). Prostatic compensation of the vitamin D axis in African American men. JCI Insight, 2(2), e91054. 10.1172/jci.insight.91054 28138564 PMC5256134

[acel13509-bib-0032] Smith, A. D. , & Refsum, H. (2016). Homocysteine, B vitamins, and cognitive impairment. Annual Review of Nutrition, 36, 211–239. 10.1146/annurev-nutr-071715-050947 27431367

[acel13509-bib-0033] Society for Adolescent Health & Medicine . (2013). Recommended vitamin D intake and management of low vitamin D status in adolescents: A position statement of the society for adolescent health and medicine. Journal of Adolescent Health, 52(6), 801–803. 10.1016/j.jadohealth.2013.03.022 23701889

[acel13509-bib-0034] Stambolsky, P. , Tabach, Y. , Fontemaggi, G. , Weisz, L. , Maor‐Aloni, R. , Sigfried, Z. , Shiff, I. , Kogan, I. , Shay, M. , Kalo, E. , Blandino, G. , Simon, I. , Oren, M. , & Rotter, V. (2010). Modulation of the vitamin D3 response by cancer‐associated mutant p53. Cancer Cell, 17(3), 273–285. 10.1016/j.ccr.2009.11.025 20227041 PMC2882298

[acel13509-bib-0035] Stine, W. B. , Jungbauer, L. , Yu, C. , & LaDu, M. J. (2011). Preparing synthetic abeta in different aggregation states. Methods in Molecular Biology, 670, 13–32. 10.1007/978-1-60761-744-0_2 20967580 PMC3752843

[acel13509-bib-0036] Sun, J. (2016). VDR/vitamin D receptor regulates autophagic activity through ATG16L1. Autophagy, 12(6), 1057–1058. 10.1080/15548627.2015.1072670 26218741 PMC4922437

[acel13509-bib-0037] Suram, A. , Venugopal, C. , Prakasam, A. , & Sambamurti, K. (2006). Genotoxicity in Alzheimer's disease: Role of amyloid. Current Alzheimer Research, 3(4), 365–375. 10.2174/156720506778249380 17017867

[acel13509-bib-0038] Tanji, K. , Miki, Y. , Ozaki, T. , Maruyama, A. , Yoshida, H. , Mimura, J. , Matsumiya, T. , Mori, F. , Imaizumi, T. , Itoh, K. , Kakita, A. , Takahashi, H. , & Wakabayashi, K. (2014). Phosphorylation of serine 349 of p62 in Alzheimer's disease brain. Acta Neuropathologica Communications, 2, 50. 10.1186/2051-5960-2-50 24886973 PMC4035093

[acel13509-bib-0039] Tuohimaa, P. (2009). Vitamin D and aging. Journal of Steroid Biochemistry and Molecular Biology, 114(1–2), 78–84. 10.1016/j.jsbmb.2008.12.020 19444937

[acel13509-bib-0040] Wang, H. X. , Wahlin, A. , Basun, H. , Fastbom, J. , Winblad, B. , & Fratiglioni, L. (2001). Vitamin B(12) and folate in relation to the development of Alzheimer's disease. Neurology, 56(9), 1188–1194. 10.1212/wnl.56.9.1188 11342684

[acel13509-bib-0041] Wu, S. , & Sun, J. (2011). Vitamin D, vitamin D receptor, and macroautophagy in inflammation and infection. Discovery Medicine, 11(59), 325–335. 21524386 PMC3285235

[acel13509-bib-0042] Zanatta, L. , Goulart, P. B. , Goncalves, R. , Pierozan, P. , Winkelmann‐Duarte, E. C. , Woehl, V. M. , & Zamoner, A. (2012). 1alpha,25‐dihydroxyvitamin D(3) mechanism of action: modulation of L‐type calcium channels leading to calcium uptake and intermediate filament phosphorylation in cerebral cortex of young rats. Biochimica et Biophysica Acta, 1823(10), 1708–1719. 10.1016/j.bbamcr.2012.06.023 22743040

[acel13509-bib-0043] Zare‐Shahabadi, A. , Masliah, E. , Johnson, G. V. , & Rezaei, N. (2015). Autophagy in Alzheimer's disease. Reviews in the Neurosciences, 26(4), 385–395. 10.1515/revneuro-2014-0076 25870960 PMC5039008

[acel13509-bib-0044] Zhong, Z. , Sanchez‐Lopez, E. , & Karin, M. (2016). Autophagy, inflammation, and immunity: A Troika governing cancer and its treatment. Cell, 166(2), 288–298. 10.1016/j.cell.2016.05.051 27419869 PMC4947210

[acel13509-bib-0045] Zierold, C. , Darwish, H. M. , & DeLuca, H. F. (1995). Two vitamin D response elements function in the rat 1,25‐dihydroxyvitamin D 24‐hydroxylase promoter. Journal of Biological Chemistry, 270(4), 1675–1678. 10.1074/jbc.270.4.1675 7829502

