# Supplementary material for: Non‐genomic rewiring of vitamin D receptor to p53 as a key to Alzheimer's disease
Source: Aging Cell. 2021 Nov 2;20(12):e13509. doi: 10.1111/acel.13509 (PMC8672786; doi:10.1111/acel.13509)
Supplement: Supplementary file 1 — Fig S1‐S8 [file ACEL-20-e13509-s001.docx]

**Supplementary Figure**

**
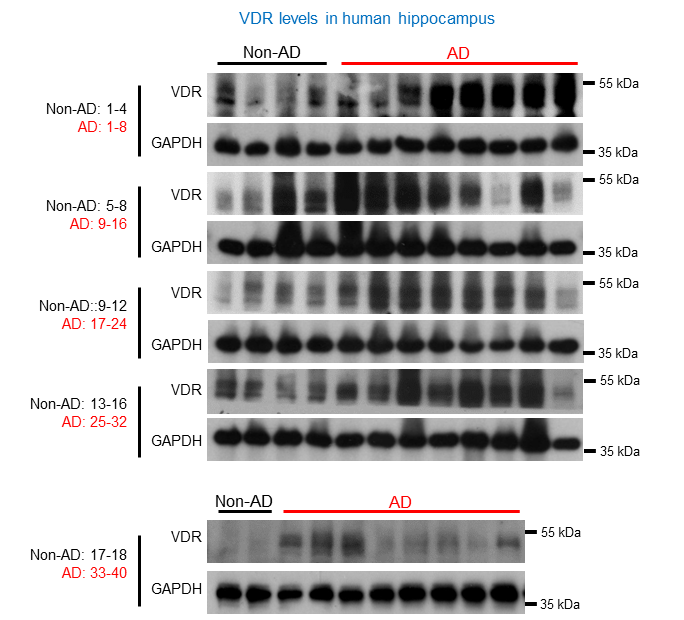
**

**Supplementary Figure 1. Elevated VDR protein levels in hippocampus tissues of AD patients.** Western blot analysis of VDR levels in hippocampus tissues of AD (n=40) patients and age and gender-matched non-AD controls (Ctrl, n=18).


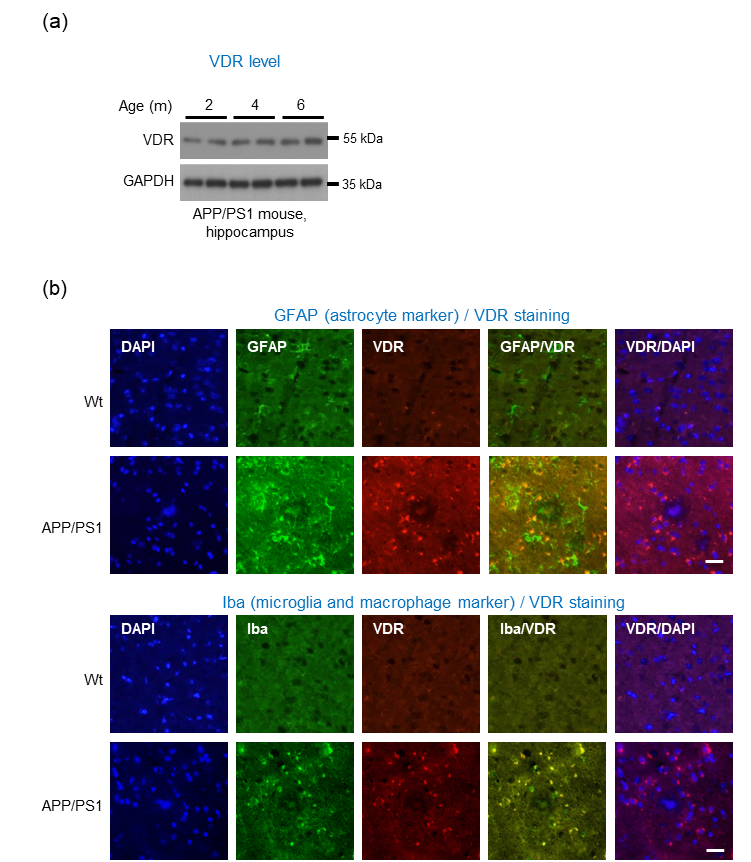


**Supplementary Figure 2. Elevated VDR protein levels in hippocampus tissues of AD patients and APP/PS1 mice.**

(a) Western blot analysis of VDR levels in the hippocampal lysates of APP/PS1 mice supplemented with cholecalciferol (inactive vitamin D_3_).

(b) Representative images of immunohistochemistry analysis for GFAP (astrocytes marker), Iba (microglia marker) and VDR in sections of Aβ plague region of hippocampus in APP/PS1 mice. Scale bars, 20 μm.

**
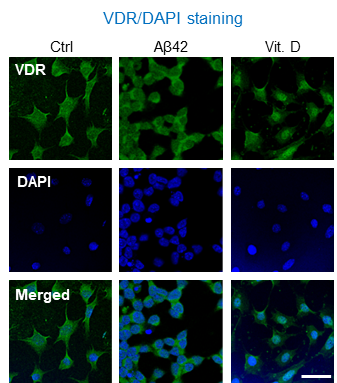
**

**Supplementary Figure 3. Representative immunofluorescence images showing cytosolic and nuclear staining of VDR in SH-SY5Y cells exposed to Aβ42.** Scale bars, 50 μm.

**
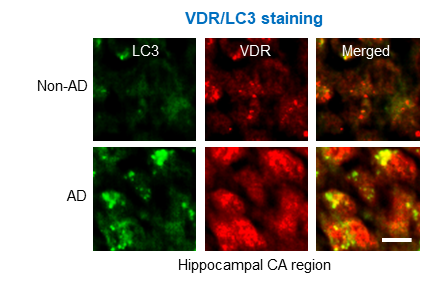
**

**Supplementary Figure 4. Representative immunofluorescent micrographs stained for VDR and LC3 in the hippocampal tissue sections of mouse AD brains.** CA regions in AD and non-AD mouse brains. Scale bars, 10 μm.

**
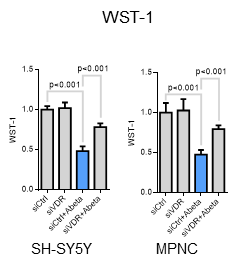
**

**Supplementary Figure 5.** **WST-1 assays for the Aβ42-induced apoptosis in cells after** **RNAi depletion of VDR.** SH-SY5Y and MPNC cells with RNAi depletion of VDR or MOCK control were treated with or without 4 μM of β-amyloid (Aβ42) for 6 h before assays. WST cell viability assay was used to determine apoptotic cell death. P < 0.05 by unpaired t-test.


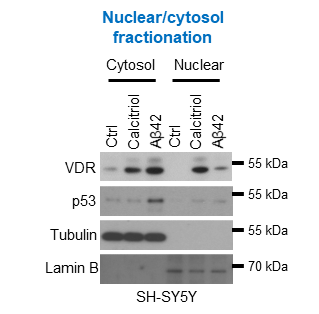


**Supplementary Figure 6. Aβ42 increases cytosol levels of p53.** SH-SY5Y cells were treated with 100 nM calcitriol or 4 μM Aβ42 for 6 h prior to assay.


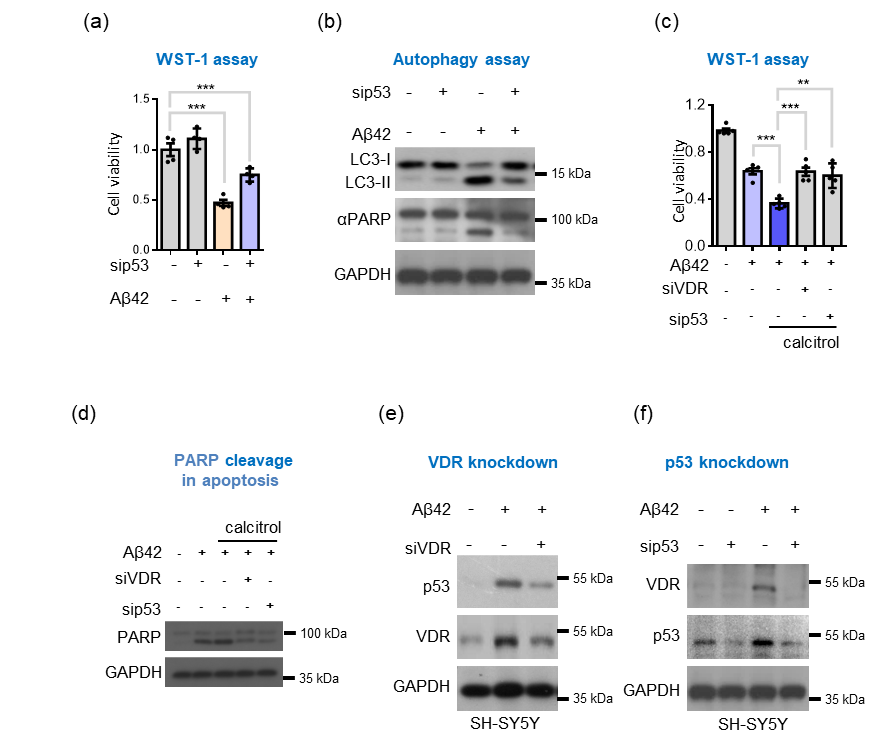
**Supplementary Figure 7. p53 modulation of the Aβ42-induced neuronal autophagy and apoptosis in neuronal cells.**

(a) WST-1 assays for the Aβ42-induced apoptosis in SH-SY5Y cells after RNAi depletion of p53.

(b) Western blot analysis of hallmarks of the Aβ42-induced autophagy and apoptosis in the p53-depleted SHS-Y5Y cells.

(c and d) Additive enhancement of apoptosis by Aβ42 and calcitriol is ameliorated by RNAi depletion of p53. SH-SY5Y cells were treated with or without 4 μM of Aβ42 plus 100 nM calcitriol for 6 h prior to WST assays (c), or western blot analysis of PARP cleavage (d). Values are represented as the mean ± SEM and *P< 0.05 by unpaired t-test.

(e) VDR and p53 protein levels in SH-SY5Y cells with or without treatment of Aβ42 and siRNA-mediated Knockdown of VDR.

(f) VDR and p53 protein levels in SH-SY5Y cells with or without treatment of Aβ42 and siRNA-mediated knockdown of p53.


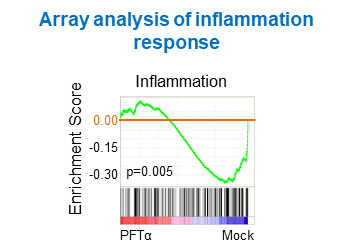


**Supplementary Figure 8. Gene Set Enrichment Analysis (GSEA) showing inflammatory response gene sets differentially expressed in APP/PS1 mice after PFTα treatment.** The inflammatory response according to the HALLMARK_INFLAMMATORY_RESPONSE gene set.
